# Mindful Embodied Movement: Study Protocol for a 12-Week Modern Dance-Mindfulness Intervention and Mixed-Methods Randomized Controlled Trial in Recreational Adult Dancers

**DOI:** 10.3390/mps9020037

**Published:** 2026-03-03

**Authors:** Aglaia Zafeiroudi, Ioannis Tsartsapakis, Charilaos Kouthouris

**Affiliations:** 1 Department of Physical Education & Sport Science, University of Thessaly, 42100 Trikala, Greece; 2 Department of Physical Education & Sport Science, Aristotle University of Thessaloniki, 62122 Serres, Greece; ioantsar@phed-sr.auth.gr

**Keywords:** community-based dance education, neuroscience, polyvagal theory, motor learning, somatic education, embodied phenomenology, psychological wellbeing, nervous system regulation, creativity, flow states, social connection

## Abstract

Recreational dance offers significant psychological well-being potential. However, traditional instruction emphasizes technique while limiting attention to nervous system development and embodied meaning-making. Despite empirical support for polyvagal theory, motor learning science, somatic education, and phenomenology, their systematic integration into unified structures is not clearly established in recreational dance contexts. This protocol integrates nervous system regulation, motor learning, and creative expression within structured Imperial Society of Teachers of Dancing (ISTD) modern dance syllabus for recreational adults. It presents a 12-week integrated dance-mindfulness intervention addressing this gap through a three-phase structure grounded in neuroscience and embodied pedagogy. The intervention comprises eight standardized components delivered weekly. The randomized controlled trial evaluates intervention effects using the Satisfaction With Life Scale (SWLS), Depression Anxiety Stress Scales-21 (DASS-21), the Mindful Attention Awareness Scale (MAAS), the Subjective Happiness Scale (SHS), and the Leisure Involvement Scale (LIS). Qualitative assessment via semi-structured phenomenological interviews (Weeks 8 and 12) and weekly journaling captures somatic awareness, nervous system resilience, technical confidence, creative expression, relational and social belonging, and embodied meaning-making. Intervention participants are expected to show significantly greater improvements compared to controls. Results will establish evidence-based practice standards for recreational dance and demonstrate neuroscience integration’s efficacy for psychological wellbeing and embodied meaning-making.

## 1. Introduction

### 1.1. Background

Recreational dance is a leisure-based movement activity. It is pursued primarily for intrinsic rewards including enjoyment, self-expression, social connection, and personal well-being, as distinguished from professional performance contexts or clinical therapeutic interventions [1,2]. Research confirms that dance interventions significantly enhance emotional regulation, reduce stress, and promote social integration in recreational adult dancers and other populations [1,3,4], while also serving as a neuroprotective activity demanding complex cognitive engagement. For those navigating psychological distress, conscious movement facilitates non-verbal emotional expression and fosters psychological safety [5].

Traditional dance instruction emphasizes proprioceptive awareness to guide and coordinate complex movements [6,7]. However, dancers often receive limited support for developing psychological awareness and holistic well-being competencies that support mental health [7]. This represents a critical gap in dance education: while dance can be a powerful medium for psychological health and embodied resilience, current training rarely integrates mental skills development with technical instruction. Research has identified the absence of standardized, integrated protocols combining these elements as a significant limitation in the field [3].

Polyvagal Theory [8,9] positions the autonomic nervous system as the foundation for social engagement and learning capacity through neuroception. Neuroception is the subconscious physiological detection and interpretation of environmental cues signaling safety or threat, independent of conscious appraisal. Neuroception operates automatically, influencing whether the nervous system moves toward social engagement or defensive dysregulation. While Polyvagal Theory has generated important insights into nervous system regulation, recent neuroscientific debate has raised questions regarding specific anatomical claims [9]. This protocol draws on PVT’s overarching framework of nervous system hierarchy and safety-based learning while remaining open to evolving neuroscientific understanding.

In dance education, the presence of a safe, co-regulated environment allows the nervous system to maintain a parasympathetic tone (ventral vagal state) while remaining capable of engaging sympathetic activation for movement without triggering dysregulation into threat states, which is a critical condition for creative expression and learning [8,10]. Evidence indicates that deliberate cultivation of the Social Engagement System (ventral vagal activation) optimizes nervous system flexibility—the organism’s capacity to dynamically regulate physiological arousal to meet environmental and task demands [9,11]. This flexibility creates conditions where technical development can occur without physiological distress or threat-based dysregulation.

Motor learning science demonstrates that dance technique mastery is enhanced when new motor information builds upon, and is compatible with, existing motor schemas, generalized rules, and parameters that guide motor behavior, thereby accelerating skill integration and transfer [12,13,14]. Achieving a state of flow (full task engagement with optimal challenge) depends on the balance between an individual’s skills and the perceived task difficulty [15,16]. However, to maintain flow while progressively increasing difficulty, the gradual titration of challenge must also consider nervous system capacity. Within neuroscience frameworks, this means ensuring participants remain within their window of tolerance, the optimal arousal zone where learning occurs without triggering threat-based defensive states [10,17], thereby avoiding both anxiety (hyperarousal) and boredom (hypoarousal), which inhibit functional learning.

Functional breathing re-education provides specific techniques for physiological regulation during movement. Practices such as nasal breathing and diaphragmatic activation assist in maintaining sympathovagal balance and supporting autonomic regulation [18]. Research on acute responses to exercise protocols demonstrates that affective experience and perceived exertion vary across different training modalities [19].

Body movement influences emotion regulation through multiple complementary mechanisms. First, physiological: intense, rhythmic movement triggers neurochemical changes in neurotransmitter (serotonin, dopamine), hormone (cortisol), and endocannabinoid levels, thereby reducing anxiety and depression [18]. Second, kinesthetic/somatic: deliberate, mindful modifications of movement quality such as weight distribution, postural adjustments, and breath-informed pacing enhance emotional states through embodied feedback loops and interoceptive awareness [18]. Third, neuropsychological: dance and music activities create flow states (full task engagement with reduced self-referential thinking) mediated by dopaminergic reward systems [20], while also enhancing creative expression, self-confidence, and social connection [21,22].

The central point to this integrated approach is “embodied learning” or “bodyfulness,” where the body is treated as the site of lived experience rather than just an instrument. This approach encourages somatic awareness and regulation, transforming dance into a holistic practice of psychological flexibility [2,3,5]. The phenomenological approach recognizes dance as a process of embodied meaning-making [23,24]. Phenomenology operates as an analytical framework that documents how participants experience neurological and kinesthetic changes during the protocol [25]. This process extends beyond individual development; according to interpersonal neurobiology theory, consciousness emerges as an embodied and relational phenomenon arising through interaction [10].

Within dance contexts, embodied self-awareness provides the foundation for developing reflective capacity. The body becomes the site where reflection originates, merged with empathic connection and fostering interpersonal presence [23]. Technical skill development does not occur in isolation but rather through sustained embodied reflection nested within collaborative movement, group improvisation, and reciprocal perspective-taking [25]. When participants engage in these relational movement experiences, where individuals retain their uniqueness while simultaneously participating in collective expression, learning is catalyzed by recognition of social interdependence and awareness that personal performance contributes to collective accomplishment [3,14]. Research on motor learning demonstrates that social-comparative feedback and collaborative contexts enhance both performance expectancies and learning outcomes [14]. Consequently, technical advancement, emotional resilience, and social integration develop synergistically within an integrated, embodied learning ecology through the coordinated engagement of individual expression, relational attunement and collective coherence [3,14].

Christensen et al. [1] identified six foundational components in recreational dance: rhythm and music, sociality, technique and fitness, connectedness, flow and mindfulness, and esthetic emotions. These components develop emotional regulation through complementary mechanisms like physiological (neurochemical shifts from rhythmic movement), kinesthetic-somatic (interoceptive awareness and embodied feedback), and neuropsychological (flow states and creative expression). Recreational dance, evaluated by how it “feels” rather than how it appears, naturally reduces stress and fosters authentic self-expression in supportive, non-competitive contexts [1]. Social synchrony through group movement engages mirror neuron systems and oxytocin-mediated bonding, while technique mastery demands complex cognitive integration. Critically, while these six components operate synergistically in lived recreational dance practice, current pedagogy rarely systematizes their intentional integration or monitors their psychological effects through rigorous empirical assessment [1].

Despite the potential of dance for well-being, the current literature reveals critical disconnects in pedagogical and neuroscientific application for recreational populations. Existing interventions lack cohesive theoretical frameworks that systematically operationalize neuroscience into dance pedagogy. As Zafeiroudi et al. [3] report, programs are often fragmented, lacking the ability to clearly bridge the gap between abstract somatic theory and concrete teaching methodology. Most concerning is the distinct lack of Randomized Controlled Trials (RCTs) targeting recreational dancers. Research predominantly focuses on clinical populations (therapy) or elite professionals (performance), leaving recreational dancers, who seek well-being and expression, without clear evidence-based guidelines [2,3]. Finally, the existing literature lacks phase-based pedagogical structures that respect the nervous system, progressing from safety establishment to optimal challenge while ensuring participants remain regulated throughout the learning process during dance class.

### 1.2. Aim and Objectives

The aim of this study is to develop and theoretically justify a comprehensive, 12-week integrated dance-mindfulness protocol grounded in polyvagal theory, motor learning science, and embodied phenomenology. The protocol systematically operationalizes the cultivation of nervous system resilience, technical dance competence, authentic creative expression, and social belonging within structured modern dance theater (Imperial Society of Teachers of Dancing-ISTD syllabus [26]) for community-based adult dancers. The study investigates whether this integrated protocol produces measurable psychological improvements compared to standard dance instruction, and whether participants report enhanced embodied meaning-making and somatic awareness through phenomenological accounts. The specific objectives are:

(1) To develop and operationalize a comprehensive, 12-week integrated dance-mindfulness protocol grounded in neuroscience, motor learning, and embodied phenomenology that systematically integrates theory with practice.
(2) To evaluate the protocol’s effectiveness using a mixed-methods RCT design comparing intervention and control groups on quantitative and qualitative outcomes.
(3) To evaluate the intervention’s effectiveness on psychological well-being and embodied experience.

Based on the theoretical framework and structure of the 12-week intervention, it is hypothesized that participants in the dance mindfulness intervention group will demonstrate significantly greater improvements than those in the control group across primary quantitative outcomes (life satisfaction, subjective happiness, psychological distress, mindful attentional capacity, and leisure involvement) and secondary qualitative outcomes (enhanced somatic awareness, nervous system resilience, technical confidence, creative expression, relational belonging, social connection and embodied meaning-making).

The present protocol is designed for community-based, non-clinical recreational dance classes rather than as a therapeutic program for trauma or other clinical mental health conditions. It targets healthy adult recreational dancers and focuses on supporting everyday psychological wellbeing in the context of regular dance classes. The pedagogical approach aims to foster nervous-system regulation to facilitate learning and flow while maintaining clear professional boundaries: instructors provide structured movement and mindfulness-based cues but do not deliver psychotherapy or intentionally explore traumatic material. If a participant experiences significant emotional distress during a session, their participation in that session is paused and they are referred to external mental health professionals for appropriate clinical support.

## 2. Experimental Design

### 2.1. Trial Design

This study has been prospectively registered on ClinicalTrials.gov (NCT07262177; brief title: Dance-Mindfulness Intervention for Well-Being in Recreational Adults-DanceMind) and approved by the institutional Research Ethics Committee (IEC, DPESS, UTH 4-2/2.4.2025; Supplementary File S8 [27]). The protocol follows SPIRIT [28,29], TIDieR [30], and CONSORT standards [31].

This is a two-parallel group randomized controlled trial (RCT). The intervention group receives a 12-week integrated dance-mindfulness protocol grounded in polyvagal theory, motor learning science, and embodied phenomenology. The control group continues regular recreational dance activities without mindfulness or somatic-reflective components. Participants are randomly allocated 2:1 (intervention: control) using computer-generated randomization with allocation concealment.

Quantitative assessments occur at baseline (T0) and post-intervention (T2 Week 12). Qualitative data collection occurs at mid-intervention (T1 Week 8) and post-intervention (T2 Week 12). This mixed-methods design permits concurrent testing of quantitative psychological outcomes (life satisfaction, subjective happiness, psychological distress, mindful attention, leisure involvement) and qualitative embodied experiences (somatic awareness, nervous system resilience, technical confidence, creative expression, relational belonging, meaning-making).

All procedures follow ethical principles outlined in the Declaration of Helsinki. Written informed consent is obtained prior to enrollment. Participants have the right to withdraw at any time without consequence. Personal data are stored securely in encrypted databases. Each participant receives a unique study code; identifying information is separated from research data. All facilitators hold CPR/First Aid certification and mindfulness training. Session checklists document adverse events. If a participant reports intense psychological distress, the study is suspended for that individual with clinical referral provided. Facilitators are trained to recognize signs of emotional dysregulation and provide immediate basic containment (quiet space, grounding breath), without attempting psychological exploration. Control group participants receive delayed access to the intervention after post-intervention assessment (Supplementary Files S1–S8 [27]).

### 2.2. Study Setting

The study will be conducted across private dance schools in Thessaly, Greece. All baseline (T0) and post-intervention (T2) quantitative assessments will be held in quiet, standardized studio environments at each participating dance school to ensure ecological validity and participant accessibility. All qualitative interviews (T1 Week 8, T2 Week 12) will be conducted in private, quiet locations at the respective dance schools. The 12-week dance-mindfulness intervention will be delivered at the designated dance studios in Thessaly region in Greece. Each studio meets the standardized environmental specifications to ensure consistent and safe delivery across sites (Supplementary File S2 [27]).

### 2.3. Eligibility Criteria

Participants for this study will be recreational adult dancers aged ≥ 18 years with a minimum of 6 months prior recreational dance experience at community dance schools. Eligible participants must demonstrate capacity for sustained moderate-intensity movement (60 min sessions) and good health with no severe musculoskeletal injuries within the past 2 weeks or intense medical conditions preventing coordinated group movement. Participants must commit to attending at least 70% of sessions (minimum 8 of 12 weeks) and baseline and post-intervention assessments, and be fluent in Greek language (written/verbal).

Exclusion criteria include: (1) active engagement in concurrent mindfulness-based or somatic-movement practices (yoga, Pilates, tai-chi, MBSR programs, etc.), which share theoretical overlap with the intervention’s mindfulness and embodied awareness components and could confound results; (2) severe psychiatric symptomatology requiring clinical psychiatric intervention (hospitalization, crisis stabilization, active suicidality). However, individuals with stable, treated psychiatric conditions are eligible, provided they have been stable on their current medication for a minimum of 6 months and have medical clearance from their treating clinician confirming appropriateness for moderate-intensity physical activity; (3) active substance abuse or dependence (current or within the past 12 months); (4) significant uncorrected hearing or vision impairment affecting the ability to participate in group dance and receive verbal or visual instructions; (5) current pregnancy; (6) acute physical contraindication to movement, including intense musculoskeletal injury occurring within 2 weeks prior to enrollment, severe infection, or uncontrolled cardiovascular event.

Individuals with injuries, mild to moderate, well-controlled chronic medical conditions (e.g., diabetes, hypertension, asthma, arthritis) or stable chronic psychological conditions are not exclusionary if participants provide written medical clearance from their treating physician/clinician confirming capacity for sustained moderate-intensity movement (90–100 min sessions) and documenting any activity modifications required. Severe or poorly controlled chronic conditions are exclusionary and require individual assessment by the principal investigator in consultation with the participant’s clinician.

During preliminary telephone or in-person screening, trained site coordinators conduct structured interviews assessing adherence to inclusion and exclusion criteria. Prospective participants receive a comprehensive eligibility information sheet listing all criteria in accessible language and are invited to self-identify any exclusions or concerns. Those meeting preliminary eligibility criteria (age ≥ 18, minimum 6 months recreational dance experience, availability to attend ≥70% of sessions, Greek language fluency) proceed to formal baseline assessment. Site coordinators maintain a screening log documenting all preliminary screenings, including numbers screened, numbers excluded at each stage, and reasons for exclusion, to enable identification of potential selection bias.

### 2.4. Sample Size and Recruitment

To determine the appropriate sample size for this study, a power analysis was conducted using GPower 3.1 software [32]. The analysis was based on a repeated-measures design with two groups (experimental and control), with measurements taken at baseline and post-intervention. The following parameters were used: Effect Size (Cohen’s d) = 0.50 (medium effect); Alpha Level (α) = 0.05 (5% significance threshold); Power (1 − β) = 0.80 (80% probability of detecting a true effect); two measurement time points (pre- and post-intervention); and two groups (experimental and control). According to the power analysis, the minimum sample size required to achieve sufficient statistical power is 114 participants, with 38 participants in the control group and 76 in the experimental group.

Participants will be informed about the study through targeted outreach at private recreational dance schools in four cities (Larissa, Trikala, Karditsa, Kalampaka) within the Thessaly region, Greece, with an estimated 1–2 participating schools across these cities. Recruitment methods include flyers, posters, verbal announcements during dance classes, and direct recruitment by dance school directors. Recruitment occurs over an anticipated 1–2 month period, with baseline assessments completed by the end of Month 2. During initial contact, prospective participants receive a participant information sheet detailing study aims, procedures, risks, benefits, confidentiality protections, and withdrawal rights. The information sheet describes the study as “a 12-week integrated modern dance program” without mentioning mindfulness components, to minimize selection bias toward mindfulness-seeking participants. Individuals expressing interest undergo preliminary telephone or in-person screening with a trained site coordinator to assess adherence to inclusion and exclusion criteria (Section 2.3). Those meeting preliminary eligibility criteria are informed of the next steps. Individuals with intense mental health distress or active engagement in concurrent mindfulness-based interventions are offered future participation following symptom resolution or completion of conflicting interventions, rather than immediate exclusion.

Participants proceed to formal baseline assessment (T0), which includes informed consent and administration of quantitative outcome measures (SWLS, DASS-21, MAAS, SHS, LIS; approximately 20–30 min). After completion of baseline assessment (T0), eligible participants are randomly allocated 2:1 (intervention: control) using computer-generated randomization with allocation concealment (administered by an independent data manager not involved in recruitment or assessment). Randomization is conducted via a secure, password-protected electronic system accessible only to the data manager, ensuring that recruitment staff and outcome assessors remain blind to allocation. Following randomization, intervention group participants receive full disclosure of the 12-week dance-mindfulness protocol and provide additional written informed consent confirming understanding of the intervention and commitment to attending the sessions.

Control group participants receive written guidelines regarding their usual recreational dance activities during the 12–week study period, including instructions to exclude concurrent mind–body practices (yoga, Pilates, tai-chi, MBSR programs, etc.). Control group participants are informed that they will have access to the full 12-week dance-mindfulness program at no cost after post-intervention assessment (T2, Week 12), providing equitable delayed intervention access.

Site coordinators maintain a screening log documenting all preliminary screening contacts, including date of screening, recruitment source, preliminary eligibility status (age, dance experience, attendance availability, language fluency), exclusion criteria met (if applicable, specifying which criterion 1–6), clearance documentation status, and disposition (enrolled in baseline assessment, not meeting criteria, awaiting clearance). This log enables complete CONSORT [31] flowchart documentation and allows identification of potential selection bias or recruitment barriers. In the event that randomized participants withdraw or fail to meet attendance criteria (≤70% of sessions), they are retained in all analyses using intention-to-treat methodology. Missing outcome data will be addressed through multiple imputation or last-observation-carried-forward approaches, as appropriate based on data missingness patterns. Replacement participants are not recruited mid-trial, as this would create methodological complications (unbalanced final sample size, potential confounding from replacement timing differences). Final sample size analyzed may differ from target sample size and will be fully documented in the CONSORT compliant participant flowchart, which will be generated upon study completion detailing actual enrollment, randomization, and attrition data by group and reason for withdrawal.

All participants provide written informed consent prior to baseline enrollment. Personal data are handled in strict compliance with the General Data Protection Regulation. All participant identifiers are replaced with unique numeric codes; codebooks linking codes to identifying information are encrypted and stored separately from all research data, accessible only to the principal investigator. Paper materials are stored in locked filing cabinets in restricted-access laboratory space; digital data are stored on secure, password-protected institutional servers with automated daily backups. Data are retained for five years following study completion; paper documents are shredded, and digital data securely deleted thereafter, in accordance with institutional policies and GDPR regulations.

### 2.5. Measurement Instruments

All quantitative outcome measures are validated for Greek adult populations and administered at baseline (T0) and post-intervention (T2). Qualitative data collection occurs at mid-intervention (T1, Week 8) and post-intervention (T2, Week 12).

The quantitative measurement instruments are presented in the following Table 1.

The qualitative data collection instruments will be the following (Supplementary File S3 [27]):

Phenomenological Interview Guide. A semi-structured interview protocol guides qualitative data collection at mid-intervention (T1, Week 8, *n*~30) and post-intervention (T2, Week 12, *n*~30). The interview guide contains open-ended questions focused on: (1) embodied experience and somatic awareness (e.g., “Can you describe moments when you felt particularly aware of your body during the sessions?” and “How did your relationship with your breath or bodily sensations evolve across the weeks?”); (2) emotional awareness and regulation (e.g., “What emotional experiences stood out for you during the program?”); (3) movement identity and creative expression (e.g., “How has your relationship with dance or your own movement changed?”); (4) meaning-making and personal insights (e.g., “What meaning did you make from your experiences in the program?” and “Were there moments that connected with your personal life, values, or self-understanding?”); (5) perceived changes like psychological, physical, and social (e.g., “Since the beginning of the program, what changes, if any, have you noticed in yourself?” and “Do you feel different in daily life because of this experience?”); and (6) program structure and safety (e.g., “How did you experience the environment and safety of the sessions?”). Interviews are digitally recorded (after participant consent) and transcribed verbatim for qualitative analysis.

Weekly Journaling Prompts. Participants provide weekly written reflections using standardized prompts distributed at the end of each session (5–10 min per entry). Prompts guide attention to: (1) breath and embodiment (e.g., “Which movements or exercises helped you feel embodied or aware of your internal sensations?”); (2) emotional states and internal reactions (e.g., “Did you notice any shifts in mood, tension, or energy throughout the session?”); (3) awareness of movement and creative exploration (e.g., “Were there moments where movement felt effortless, challenging, playful, or meaningful?”); (4) meaning-making (e.g., “How did today’s experience connect with your life outside the studio?”); and (5) change across sessions (e.g., “Compared to earlier weeks, do you notice any changes in your awareness, emotions, or movement?”). Journals are completed on standardized paper forms, collected weekly, and analyzed for thematic patterns regarding embodied experience, affective change, and perceived transformation.

### 2.6. Intervention and Control Conditions

The intervention group will consist of participants who attend 12 weekly sessions (90–100 min each) structured across three phases: (1) Foundations (Weeks 1–4), (2) Embodied Exploration (Weeks 5–8), and (3) Expressive Integration (Weeks 9–12). Each session integrates eight standardized components (warm-up/breath grounding, technical dance work, conditioning, mindful break, rhythm/improvisation, choreographed sequences, cool-down/reflective integration, phenomenological journaling). Detailed component descriptions, facilitator language, and session structure are specified in Supplementary File S1 [27].

The control group will consist of participants who continue usual recreational dance activities (preferred modern dance and/or contemporary classes) for 12 weeks, excluding mind–body practices (e.g., yoga, Pilates, tai-chi) that share overlap with the intervention. Control participants do not receive mindfulness training, somatic cuing, or reflective journaling prompts. After post-intervention assessment (T2, Week 12), control participants are offered access to the full 12–week Dance-Mindfulness program at no cost (delayed intervention, ethical equity control).

### 2.7. Data Collection and Analysis

Self-report questionnaires (SWLS, DASS-21, MAAS, SHS, LIS) are administered on paper at baseline (T0) and post-intervention (T2) by trained site coordinators in quiet studio environments (~20–30 min per assessment). Questionnaires are entered into a secure electronic database with double-entry verification.

Demographic and baseline characteristics are summarized using descriptive statistics (means, standard deviations, medians, frequencies, percentages). Shapiro–Wilk tests and visual inspection (Q-Q plots, histograms) assess normality for all primary outcome variables. Between-group demographic comparisons use independent-samples *t*-tests (continuous) and chi-square tests (categorical). Internal consistency is assessed using Cronbach’s alpha (α ≥ 0.70); test–retest reliability via intraclass correlation coefficients (ICC ≥ 0.70) in the control group.

If normality is met (Shapiro–Wilk *p* > 0.05), a 2 (Group) × 2 (Time) mixed-model repeated-measures ANOVA is conducted on each primary outcome, with Bonferroni-corrected α = 0.01 to control Type I error. Alternative testing via MANOVA (Wilks’ Lambda, α = 0.05) proceeds to univariate ANOVAs if significant. ANCOVA with T0 baseline covariates is conducted with Levene’s test assessing homogeneity of variance; if violated (*p* < 0.05). Effect sizes and 95% confidence intervals are reported.

If normality is violated (Shapiro–Wilk *p* < 0.05), non-parametric tests will be used: Friedman’s test (within-group time effects), Mann–Whitney U (between-group comparisons), Wilcoxon signed-rank test (within-group pre–post). Post hoc pairwise comparisons use Bonferroni-corrected alpha. Pearson correlations (Bonferroni-corrected) for normally distributed variables; Spearman’s rank-order correlations for non-normal variables. Correlations ≥ |0.30| are highlighted. Quantitative analyses use IBM SPSS v. 28; results are reported per CONSORT guidelines [31] with summary statistics, 95% confidence intervals, and effect sizes. Significance level: α = 0.05 (two-tailed) unless otherwise specified (Bonferroni-corrected α = 0.01).

In qualitative analysis, semi-structured interviews (*n*~30, T1 Week 8 and T2 Week 12; maximum variation sampling) and weekly journal entries are collected from intervention participants. Interview data are analyzed using Interpretative Phenomenological Analysis (IPA; six-step procedure per [23,40,41] with inter-rater reliability assessed via Cohen’s kappa (κ ≥ 0.70). Weekly journals are analyzed using Reflexive Thematic Analysis [42,43] with phase-specific coding (Weeks 1–4, 5–8, 9–12). Two independent coders conduct all qualitative analysis; discrepancies are resolved through consensus. Analysis examines themes related to embodied awareness, emotional regulation, nervous system resilience, relational presence, and personal transformation (detailed methodology in Supplementary File S3 [27]).

Quantitative measures (both groups, T0–T2) test intervention efficacy; qualitative data (intervention group only) explores embodied mechanisms of change, providing mechanistic understanding complementary to quantitative hypothesis testing.

## 3. Procedure

**Figure 1:**
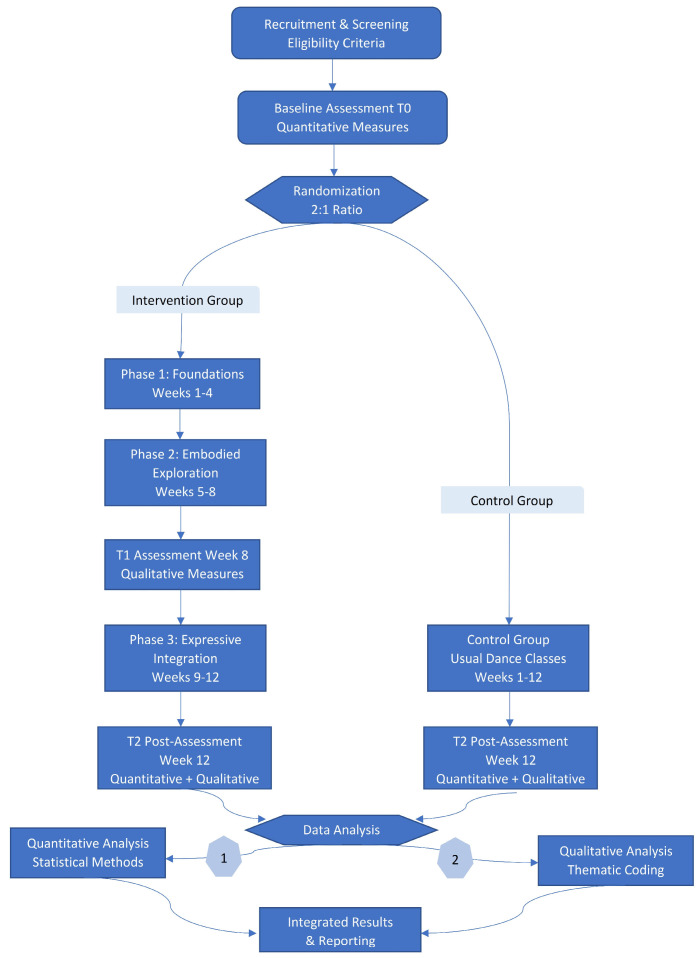
Study workflow: participant enrollment, randomization, intervention delivery, data collection timeline, and follow-up across the 12–week trial.

The study procedure intervention delivery, and data collection processes across the 12-week trial. All detailed facilitator guidelines, environmental specifications, and data management protocols are documented in Supplementary Files S2 [27]. This section provides a procedural overview; complete procedural specifications appear in Supplementary Materials (Figure 1). Data collection will begin in early January 2026 and conclude in early April 2026. Participant recruitment will be completed by 10 January 2026.

Figure 1 presents the complete study workflow across the 12-week trial. It illustrates participant flow from recruitment and baseline assessment (T0) through randomization (2:1 intervention:control), the three-phase intervention structure, mid-point qualitative assessment (T1, Week 8), post-intervention assessments (T2, Week 12), and final mixed-methods analysis combining quantitative and qualitative outcomes.

### 3.1. Intervention Delivery and Safety Monitoring

Intervention sessions are delivered weekly by trained facilitators holding CPR/First Aid certification and formal mindfulness training (qualifications detailed in Supplementary File S2 [27]). Facilitators maintain standardized session structure per Section 2.6 and detailed protocols in Supplementary File S1 (Intervention Manual) [27]. Attendance and adverse events are documented on standardized checklists completed immediately post-session.

If a participant reports severe psychological distress or adverse effects during a session, the facilitator pauses the activity, provides immediate support in a private setting, and offers clinical referral if indicated. Participation may be suspended pending further evaluation. All safety incidents are documented on incident forms and reported to the principal investigator within 24 h. If a participant reports acute psychiatric symptoms requiring hospitalization, crisis stabilization, or active suicidality, the study is suspended for that individual with clinical referral provided to a local mental health resource.

Intervention fidelity is monitored through: (1) Standardized session checklists documenting component completion, duration, deviations, and engagement level (completed immediately post-session by facilitator); (2) facilitator reflection journals documenting session flow, group atmosphere, facilitator reflections, adaptations, and concerns (reviewed weekly by Principle Investigator-PI); (3) audio recording of approximately 5–10% of sessions (stratified across phases and facilitators) for independent fidelity assessment using structured fidelity observation sheets (detailed in Supplementary File S4 [27]); and (4) monthly facilitator supervision meetings (2 h per month minimum) reviewing fidelity data, addressing participant safety, discussing facilitator well-being, and documenting protocol adaptations. Fidelity assessment occurs at three time points per site (Week 2, Week 6, Week 10) to capture early, mid-course, and late-stage implementation quality.

### 3.2. Data Collection

Quantitative outcome measures (described in Section 2.5) are administered on paper at baseline (T0) and post-intervention (T2, Week 12). Post-intervention assessments are conducted by trained coordinators/assessors blinded to group allocation; blinding is maintained by: (1) providing assessors only the participant study code (no group information); (2) restricting assessor access to baseline data or randomization records; and (3) ensuring assessors have no knowledge of intervention attendance or session fidelity ratings. Completed questionnaires are entered into a secure electronic with double-entry verification. Missing data patterns are examined; Section 2.7 specifies handling strategies.

Weekly Journaling (Intervention Group Only): Intervention group participants complete standardized written reflections (5–10 min) at the end of each session using prompts addressing: breath/embodiment, emotional states, movement awareness, meaning-making, and perceived changes across weeks (prompts detailed in Supplementary File S3 [27]). Journal forms are collected weekly by facilitators and maintained in secure storage. A purposive sample of journal entries (selected from Weeks 1, 4, 8, and 12, plus entries with maximum phenomenological variation) is analyzed for thematic patterns regarding embodied experience, affective change, and perceived transformation using Reflexive Thematic Analysis with phase-specific coding (Weeks 1–4, 5–8, 9–12; detailed methodology in Supplementary File S3 [27]). Two independent coders will analyze qualitative data using structured coding protocols; differences are resolved through consensus discussion. Control group participants do not complete journaling; they complete quantitative assessments only (T0 and T2).

Semi-Structured Interviews (Intervention Group Subsample): Approximately 30 intervention group participants are selected via maximum variation sampling (Section 2.7). Semi-structured interviews are conducted by trained research staff not involved in intervention delivery at mid-intervention (T1, Week 8) and post-intervention (T2, Week 12) in private, quiet locations at the respective dance schools. Each interview is approximately 20–30 min in duration and is audio recorded after participant consent. The interview guide (Supplementary File S3 [27]) addresses: embodied experience and somatic awareness, emotional awareness and regulation, movement identity and creative expression, meaning-making, perceived changes (psychological, physical, social), and program structure and safety.

Interview data will be analyzed using Interpretative Phenomenological Analysis (IPA) following the six-step procedure [40]: (1) reading and re-reading transcripts; (2) initial line-by-line coding; (3) developing emergent themes within each case; (4) searching for connections across themes within cases; (5) repeating steps 1–4 for each interview independently; and (6) cross-case analysis to identify superordinate themes and patterns (Supplementary File S3 [27]).

### 3.3. Study’s Intervention Protocol

The 12–week dance mindfulness intervention was structured as a phased, component-based protocol delivered through weekly 90–100 min sessions, organized across three developmental phases (Phase 1: Foundational Weeks 1–4; Phase 2: Embodied Exploration Weeks 5–8; Phase 3: Expressive Integration Weeks 9–12) and comprising eight standardized components designed to integrate nervous system regulation, motor skill development, and embodied meaning-making within ISTD modern dance syllabi [26]. Each session follows the standardized eight-component protocol detailed in Supplementary Files S1 [27]:

1. Warm-up and breath grounding (9–10 min);
2. Technical dance work (14–15 min);
3. Conditioning and strength work with nervous system awareness (9–10 min);
4. Break/transition and nervous system settling (4–5 min);
5. Rhythm and improvisation (8–9 min);
6. Choreographed dance sequences (15–17 min);
7. Cool-down and reflective integration (8–9 min);
8. Phenomenological journaling (10–20 min, flexible duration).

Component 1: Warm-Up and Breath Grounding (9–10 min).

Component 1 is a 9–10 min warm-up establishing embodied presence and nervous system readiness through progressively complex breath-movement integration across three four-week phases. Phase 1 (Weeks 1–4) establishes parasympathetic baseline through synchronized nasal breathing (4-count pattern) with gentle arm movements, light rhythmic activation, and joint mobility sequences. Nasal breathing activates nitric oxide production in paranasal sinuses, enhancing oxygen utilization and reducing chemosensitivity to CO_2_, key mechanisms for parasympathetic engagement [18]. Joint mobility and sensory-kinetic awareness develop interoceptive capacity, allowing participants to access sources of evaluation based on felt sensation rather than external perfectionistic criteria [44]

Phase 2 (Weeks 5–8) develops sympathetic–parasympathetic balance by adding coordinated leg extensions, multi-directional stepping (invisible cross patterns), and elevated-heart-rate activities while maintaining nasal breathing. This trains the nervous system to sustain sympathetic mobilization while maintaining parasympathetic baseline, a critical resilience capacity [8,18]. Learning strategies embedded in facilitator cuing support autonomy and reduce perfectionistic cognitions [44].

Phase 3 (Weeks 9–12) achieves embodied automaticity by weaving all movements into one continuous 7–8 min sequence. Participants execute complex motor patterns automatically while maintaining nervous system coherence, demonstrated by steady heart rate, relaxed presence, and uninterrupted nasal breathing. Dance and movement are recognized as neural exercises that shift affective states within a safe context, optimizing mental and physical health [45].

The three-phase progression mirrors motor learning stages [14]: cognitive (Phase 1, conscious effort), associative (Phase 2, coordinated integration), and automatic (Phase 3, effortless execution) (Table 2). Facilitator language continuously signals safety via warm presence and explicit nervous system cuing, activating the social engagement system and supporting parasympathetic dominance throughout [8]. This approach operationalizes vagal efficiency, the dynamic effectiveness of the vagal brake in regulating heart rate and metabolic output to match environmental and situational demands [8,45].

Component 2: Technical Dance Work (14–15 min)

Phase 1 (Grade 2) establishes body literacy (the ability to identify and reference internal sensation) creating an interoceptive foundation for technical development [46]. Dancers learn basic patterns while optimizing motor synergies and reducing unnecessary muscle tension [47]. Phase 2 (Grade 3) deepens proprioceptive awareness through enhanced complexity, operationalizing how explicit and implicit motor sequence learning occur simultaneously [12,13]. Participants consciously recall movements while implicit systems optimize motor efficiency. Facilitator cuing emphasizes interoceptive feedback rather than external form correction [46]. Phase 3 (Grade 4) integrates embodied self-awareness into authentic expression [23]. Complex sequences become vehicles for the dancer’s voice. Transfer learning principles enable Grade 3 to Grade 4 compatibility through shared movement transitions [13], demonstrating that technical mastery emerges from interoceptive awareness, not perfectionistic replication [23] (Table 3).

Component 3: Conditioning and Strength Work (9–10 min).

Phase 1 (Weeks 1–4) develops foundational embodied awareness during light exertion, teaching the nervous system that effort and parasympathetic presence coexist. Participants learn to breathe through work rather than against it, establishing an interoceptive baseline where sensation-awareness precedes physical adaptation [48]. Phase 2 (Weeks 5–8) deepens nervous system flexibility through rhythmic endurance and oscillation between effort and recovery, demonstrating how the body can sustain challenge while maintaining regulatory access [9]. Participants consciously regulate intensity via breath-monitoring while implicit motor systems optimize efficiency. Facilitator cuing shifts from “work harder” to “what is your breath telling you?” Phase 3 (Weeks 9–12) integrates vagal efficiency (the capacity to meet complex demands while maintaining parasympathetic access) through sustained whole-body effort under controlled intensity [9]. Technical strength becomes clear, serving embodied resilience and nervous system coherence [49]. Participants experience, directly, that they can sustain intensity while staying regulated; this shift in neuroception (the nervous system’s implicit detection of safety) is the core mechanism of trauma recovery [9,48] but, here, it enables optimal flow states in recreational movement practice (Table 4).

Component 4: Nervous System Settling and Break (4–5 min).

Phase 1 (Weeks 1–4) establishes parasympathetic reactivation following moderate exertion, teaching the body that intensity naturally resolves into calm. Participants practice sustained parasympathetic engagement through gentle grounding, developing interoceptive confidence in the nervous system’s capacity to self-regulate downward [17]. Phase 2 (Weeks 5–8) deepens parasympathetic recovery through rhythm and vagal tone restoration, demonstrating the nervous system’s oscillatory capacity and the transition from sympathetic arousal to parasympathetic dominance [11]. Heart rate variability (HRV) returns toward baseline as recovery progresses; facilitator cues emphasize interoceptive confirmation of this shift (“Feel your heartbeat slowing… feel your breath deepening…”). Phase 3 (Weeks 9–12) integrates embodied belonging and collective nervous system coherence through co-regulated presence, where participants experience their individual nervous system settling within the group’s shared regulation [10]. This “feeling felt” by the group reinforces the ventral vagal pathway and establishes relational nervous system healing. Recovery is complete when participants’ interoceptive baseline has reset and parasympathetic dominance is confirmed via visible calm and stable breath [19,50] (Table 5).

Component 5: Rhythm and Improvisation (8–9 min).

Phase 1 (Weeks 1–4) introduces foundational rhythm awareness through structured, minimally improvised movement, establishing intrinsic motivation through novelty and autonomy. Participants explore rhythm within contained parameters, developing felt sense of pulse and timing while maintaining a clear skill-challenge balance [51]. Phase 2 (Weeks 5–8) deepens flow state conditions through guided improvisation with increasing creative autonomy, where implicit learning systems optimize movement generation while attention focuses on embodied expression. Flow emerges when challenge and skill are balanced at moderate-to-high levels; participants experience loss of self-consciousness and merging of action-awareness [20]. Phase 3 (Weeks 9–12) integrates full improvisational engagement with optimally challenging creative tasks, where flow becomes dominant and participants experience absorption, full task engagement with low self-referential thinking [22,51]. Improvisation serves as both an anxiety-reduction tool and a pathway to embodied authenticity; through voluntary creative choices, participants access intrinsic motivation and experience the cascade of neurochemical states (dopamine, norepinephrine activation) that accompany sustained flow [20,21] (Table 6).

Component 6: Choreographed Dance Sequences (15–17 min).

Phase 1 (Weeks 1–4) introduces foundational choreographic learning through motor sequence acquisition, where declarative knowledge (step-by-step instructions) builds individual competence and somatic confidence. Participants develop beta oscillations in motor cortices associated with early learning; movement remains cognitively demanding but increasingly fluent [12]. Phase 2 (Weeks 5–8) deepens motor sequence consolidation through small-group collaborative movement, where implicit learning systems bind individual elements into skilled behavior while attention transitions from “how” to “why” and “with whom.” Ensemble presence emerges as participants experience synchronized nervous systems, inter-brain resonance where individual regulation supports collective coherence [9]. Phase 3 (Weeks 9–12) integrates full choreographic mastery with embodied authenticity, where automaticity permits genuine relational presence and ensemble artistry. Participants experience somatic knowledge acquired through 8 weeks of integrated practice; movement becomes “second nature”, a manifestation of the seven attitudinal factors of mindfulness (non-judging, patience, beginner’s mind, trust, non-striving, acceptance, letting go) embedded in choreography [52]. Ensemble becomes the vehicle for experiencing collective flow, where individual skill-challenge balance within group synchronization produces optimal task engagement and merging of action-awareness [12,15] (Table 7).

Component 7: Cool-Down and Reflective Integration (8–9 min).

Phase 1 (Weeks 1–4) establishes foundational cool-down through gentle movement and guided body scan, introducing participants to basic interoceptive awareness and parasympathetic settling. The body scan meditation, attending to sensations from feet to crown, activates the “sixth sense” (vertical integration via interoception) while facilitating memory consolidation of the session’s somatic learning through systematic attention to embodied sensation [52,53]. Phase 2 (Weeks 5–8) deepens cool-down through expanded stretching and reflective embodied awareness, where participants develop capacity to notice both sensations and emotions present in the body. Embodied reflection (attending to body phenomenologically through Merleau-Ponty’s lens) reveals how the body is the site where consciousness meets world, where hidden assumptions about our experience can surface through careful, embodied attending [54]. Phase 3 (Weeks 9–12) integrates full reflective embodied practice, where participants experience the seven attitudinal factors of mindfulness through cool-down: non-judging (observing sensations without evaluation), patience (allowing release to unfold), beginner’s mind (approaching body anew each session), trust (in natural settling), non-striving (releasing effort), acceptance (of what arises), and letting go (of tension and narratives) [52]. (Table 8) Neural and relational integration mutually support one another; as participants consolidate somatic memories through body scan and reflective practice, their individual nervous system integration simultaneously enables interpersonal regulation and collective well-being [10,53].

Component 8: Phenomenological Journaling (10–20 min).

Phase 1 (Weeks 1–4) introduces foundational narrative-emotional meaning-making through guided journaling prompts focused on individual nervous system learning: “What did your body discover today?” Participants develop capacity to construct coherent narratives of their sensorimotor experience; writing consolidates learning through repeated re-representation of embodied events as language [55]. Phase 2 (Weeks 5–8) deepens journaling to include relational and emotional integration, where narrative-emotion process markers emerge: participants move from “Same Old Storytelling” (habitual narrative) through “Inchoate Storytelling” (tentative new narratives) toward “Discovery Storytelling” (transformed meaning-making) [56]. Phase 3 (Weeks 9–12) integrates full phenomenological reflection across four dimensions: individual transformation, relational attunement, spatial inhabitation, and social belonging. Participants practice embodied reflective practice, attending to how esthetic experience lived through movement translates into articulated meaning through written reflection [25,57]. Journaling serves as both memory consolidation mechanism (translating implicit motor learning into explicit declarative memory) and meaning-making vehicle where participants construct coherent narratives of their developing sense of self through repeated cycles of embodied experience and reflective articulation [55,58] (Table 9).

### 3.4. Data Security and Management

All quantitative data will be entered into a secure, password-protected electronic database by trained data entry personnel, with range and consistency checks applied. Qualitative interviews are transcribed verbatim by trained transcriptionists, and transcripts are reviewed for accuracy against the audio recording. All participant identifiers are replaced with unique numeric codes; a codebook linking names to participant IDs is encrypted and stored separately from all data, accessible only to the principal investigator.

Paper questionnaires and journaling material are stored in restricted-access, locked filing cabinets at a research laboratory of Applied Leisure Sciences at the Department of Physical Education and Sport Science, University of Thessaly. Only the principal investigator and authorized research assistants have access. All data will be retained for five years following study completion; paper documents are shredded, and digital/audio data are securely deleted thereafter, in accordance with institutional data retention policies and GDPR regulations.

### 3.5. Intervention Facilitator Qualifications and Training

Intervention sessions are delivered by certified modern dance educators meeting the following minimum qualifications: (1) bachelor’s degree or equivalent certification in dance or somatic movement; (2) minimum two years prior teaching experience with recreational adult populations; (3) formal training in mindfulness-based practices (MBSR certification, 200 h yoga teacher training, or safety-aware somatic training). Trauma-informed training is optional for facilitators with relevant background but not required, as the protocol maintains clear pedagogical boundaries; and (4) current CPR/First Aid certification. Prior to participant contact, each facilitator completes a 12 h standardized training module based on the Intervention Protocol Manual (Supplementary S1 [27]) and participates in 1–2 mock teaching sessions observed by the principal investigator, with written feedback incorporated before first participant session delivery.

Prior to each session, facilitators complete environmental standardization checklists documenting: studio temperature (17–22 °C), illumination (300–500 lux, LED flicker-free), acoustic baseline (≤50 dB), floor conditions (sprung or semi-sprung surface), room dimensions and participant spacing (3.5–4.0 m^2^ per participant), and confirmation of clear emergency exit pathways and visible first aid kit location (detailed specifications in Supplementary File S2 [27]).

Session attendance is recorded by the facilitator at each weekly session. If a participant misses a session, the site coordinator contacts them within 48 h to understand reasons and reschedule a make-up session if feasible, preferably within the same phase. Participants missing more than two consecutive sessions receive additional follow-up contact to assess barriers and provide support (childcare resources, transportation assistance, schedule accommodation).

Facilitators complete standardized fidelity checklists immediately post-session documenting: completion of all eight components, actual duration of each component, deviations from protocol with justification, participant engagement level, and any adverse events or clinical concerns. Facilitators also maintain a reflective journal after each session documenting session flow, participant engagement, emotional atmosphere, personal reflections on facilitation quality, adaptations made, and any participant concerns. Reflection journals are reviewed weekly during supervision.

Facilitators meet with a designated supervisor (principal investigator or designated research staff) for weekly 1 h supervision sessions (or biweekly if multiple facilitators). Supervision includes fidelity review, problem-solving regarding participant responses or challenges, discussion of participant safety concerns, facilitator well-being and support needs, and any protocol adaptations with documented justification.

### 3.6. Mid-Intervention Qualitative Assessment (T1, Week 8)

At Week 8, qualitative data collection captures participants’ lived experience at the intervention midpoint, creating a comparative baseline for the post-intervention assessment. A purposive subsample of approximately 30 intervention participants is selected via maximum variation sampling (varying by age, gender, prior experience, and baseline distress). These participants complete a 20–30 min interview exploring six core phenomenological domains (embodied awareness, emotional regulation, nervous system activation, relational connection, meaning-making, and movement confidence). The interview guide (Supplementary File S3) [27] specifically probes shifts in embodied experience attributed to the first two phases (Foundations and Embodied Exploration), allowing for longitudinal tracking of the learning trajectory.

Cumulative weekly journals from Weeks 1 to 8 are collected to form the Phase 1 and Phase 2 dataset. Unlike retrospective interviews, these entries provide real-time, week-by-week accounts of the embodied learning process before the onset of the final Expressive Integration phase (full journaling prompts available in Supplementary File S3 [27]). Control group participants do not engage in interviews or journaling.

### 3.7. Post-Intervention Assessment (T2, Week 12)

At Week 12, all randomized participants complete the final assessment to evaluate intervention outcomes and longitudinal progression. Both intervention and control groups complete the identical battery of five outcome measures administered at baseline (T0), following standard administration procedures to ensure comparability. The same subsample of participants interviewed at T1 completes a second semi-structured interview (20–30 min). The T2 guide (Supplementary File S3) [27] retains the core phenomenological domains but pivots the inquiry toward integration and transformation. Participants reflect on their trajectory across the full 12-week period, enabling an analysis that compares T1 (midpoint) and T2 (endpoint) narratives to map the developmental arc from early somatic grounding to late-phase creative expression.

Intervention participants complete a final journal entry (Supplementary File S3) [27] specifically designed to export a retrospective comparison between their Week 1 and Week 12 embodied states. This completes the qualitative dataset, which is then stratified into three Phase-aligned segments (Weeks 1–4, 5–8, 9–12) for Reflexive Thematic Analysis (coding framework detailed in Supplementary File S3 [27]).

The qualitative strategy at T2 focuses on triangulation: phase-specific journal themes are cross-referenced with longitudinal interview narratives to construct a mechanistic explanation of embodied change. Control group participants complete only the quantitative assessment, preserving the distinction between outcome evaluation (quantitative/comparative) and process evaluation (qualitative/descriptive).

## 4. Expected Results

Based on the integrated theoretical framework (polyvagal theory, motor learning science, embodied phenomenology) and the structured 12–week protocol design, the following outcomes are expected:

Quantitative outcomes. Intervention participants are expected to demonstrate significantly greater improvements from baseline to post-intervention compared with controls on primary outcome measures: increased life satisfaction and subjective happiness, decreased psychological distress, and enhanced mindful attentional capacity and leisure involvement. These improvements are hypothesized to reflect the intervention’s integration of nervous system regulation (breath-movement synchronization), embodied awareness practices, and relational belonging within the dance context.

Qualitative outcomes. Phenomenological analysis of interview and journal data is expected to reveal a progressive developmental arc across the three intervention phases. Early-phase reflections are anticipated to center on foundational somatic awareness and nervous system regulation; mid-phase reflections to incorporate relational presence and emotional attunement; and late-phase reflections to synthesize embodied learning, relational connection, and authentic expression into coherent narratives of transformation. Control group reflections are expected to remain focused on conventional dance engagement (technique, enjoyment) without the developmental trajectory or transformational narratives anticipated in the intervention group. Individual variability in response will be examined descriptively to identify participant and contextual factors influencing intervention responsiveness.

## 5. Discussion

This protocol synthesizes empirical evidence from seven converging research domains to address a significant methodological gap in recreational dance research. Harrison et al. [6] established mindfulness-acceptance-commitment feasibility in professional dancers but revealed limitations in sample size and intervention duration. Christensen et al. [1] identified six fundamental dance components but noted underdeveloped sequencing principles, motivating the present protocol’s three-phase pedagogical structure. Dwarika et al. [59] documented methodological gaps including weak standardization and reporting fidelity. Zafeiroudi et al. [3] conducted a scoping review identifying critical gaps in theoretical integration, incomplete operationalization of intervention components, limited methodological rigor, and variable fidelity monitoring. Marich & Howell [5] established that dance and music are core mechanisms through which mindfulness-based movement produces emotional, spiritual, and psychological benefits. Porges’ polyvagal theory [8,9] provides the neuroscientific foundation for nervous system safety-based learning as a prerequisite for motor skill acquisition. Schmidt et al.’s motor learning science [14] establishes that skill consolidation occurs in phases, aligning the protocol’s three-phase structure with cognitive, associative, and autonomous motor learning stages.

Although the present protocol is grounded in Harrison et al.’s [7] theoretical framework regarding mindfulness-acceptance-commitment interventions in dancers, it differs in three dimensions. The population differs in that Harrison et al. examined 16 professional ballet dancers with limited time for mindfulness practice, whereas the present protocol targets recreational dancers who are motivated by intrinsic satisfaction rather than professional demands. The intervention structure also differs, replacing six standalone 30 min sessions with 12 sessions of 90–100 min with mindfulness embedded directly within structured choreography rather than as parallel practice. The theoretical framework extends substantially by integrating polyvagal theory and motor learning science directly into pedagogical structure and using a broad spectrum of psychological and qualitative measures, and phenomenological interviews for multidimensional assessment, whereas Harrison et al. [7] implemented only the MAC model with athlete-specific measures. Where Harrison et al. [7] found no statistically significant differences attributed to small sample size and brief duration, the present protocol addresses these limitations with 114 participants and doubles the intervention length, with embedded integration expected to enhance adherence and efficacy.

Harrison et al. [7] applied an autonomous Mindfulness-Acceptance-Commitment model originally developed for professionals, whereas the present protocol embeds MAC philosophy within a substantially more comprehensive framework structured around three integrated theoretical components. Polyvagal Theory directs every movement choice and environmental perception through neuroception and the Social Engagement System as the foundation for co-regulated learning. Motor Learning Science guides progressive challenge titration within each participant’s window of tolerance through three hierarchical phases named Foundational, Embodied Exploration, and Expressive Integration, which are designed to maintain nervous system regulation while increasing technical complexity. Embodied Phenomenology operationalizes qualitative assessment through journals and semi-structured interviews incorporating Interpretative Phenomenological Analysis, targeting somatic awareness, nervous system resilience, technical confidence, authentic creative expression, relational belonging, and embodied meaning-making. Through this integration, the protocol develops MAC from a standalone psychological intervention into an indivisible structure threaded through all eight class components, with each movement explicitly linked to neuroscientific rationale and somatic awareness.

Christensen et al. [1] identified six fundamental dance components (rhythm/music, sociality, technique/fitness, connection, flow/mindfulness, esthetic emotions) but noted heterogeneous outcomes across dance styles, suggesting that components alone are insufficient without developmental sequencing. This protocol operationalizes such sequencing through a three-phase structure (Foundations Weeks 1–4, Embodied Exploration Weeks 5–8, Expressive Integration Weeks 9–12), a contribution that develops Christensen’s framework. Importantly, this phased architecture aligns with motor learning science consolidation windows: Phase 1 establishes parasympathetic foundation (cognitive motor learning stage). Based on motor learning principles [14], Phase 2 was designed to span a period optimal for motor memory consolidation, and Phase 3 operationalizes automaticity (autonomous motor learning stage). This theoretical alignment permits empirical testing of motor learning principles within dance pedagogy—a mechanistic investigation that Christensen [1] identified as lacking.

Dwarika et al. [59] identified critical reporting and methodological gaps in dance mental skills interventions: inconsistent intervention duration (6 weeks to 1 year), lack of standardization in content delivery, and weak methodological design overall. This protocol directly addresses these gaps through operationalization of the TIDieR (Template for Intervention Description and Replication) framework [27]. All eight standardized components are documented with facilitator language, duration, environmental specifications, and theoretical grounding. This represents a methodological advancement.

However, the protocol recognize uncertainty regarding polyvagal theory’s neuroanatomical foundations. While polyvagal theory [8] provides the overarching framework for nervous system safety-based learning, recent neuroscientific debate has questioned specific anatomical claims [9]. The protocol operates pragmatically: it operationalizes polyvagal principles (progressive cultivation of parasympathetic tone, nervous system flexibility, and social engagement) as a pedagogical framework, while remaining open to evolving neuroscientific understanding. This permits the protocol’s validity to rest primarily on empirical outcomes rather than neuroanatomical confirmation.

While Zafeiroudi et al. [3] identified ten promising dance-based mindfulness interventions in recreational contexts, this scoping review simultaneously revealed critical gaps: lack of systematic theoretical integration, incomplete operationalization of intervention components, limited methodological rigor in design and outcome measurement, and variable fidelity monitoring. This protocol directly addresses each gap through a theoretically grounded, methodologically rigorous, and systematically monitored approach to recreational dance-mindfulness intervention in adults.

By combining RCT design with TIDieR operationalization, theoretical integration, refined outcome measurement, and proactive fidelity monitoring, this protocol advances the field from exploratory pilots and qualitative case studies toward systematic, replicable, evidence-based dance-mindfulness pedagogy. The results will provide the empirical foundation needed to establish recreational dance-mindfulness as a validated, standardized, and scalable approach to supporting psychological well-being, embodied awareness, and authentic self-expression in community populations.

The protocol combines quantitative hypothesis testing (does the intervention improve psychological outcomes?) with qualitative phenomenological exploration (how do participants experience embodied transformation?) and qualitative data comprise semi-structured interviews analyzed via Interpretative Phenomenological Analysis (IPA) [40,41], plus weekly journaling analyzed via Reflexive Thematic Analysis [42,43] with phase-specific coding. This dual approach addresses Christensen’s [1] observation that what exactly is driving positive effects remains unclear in dance research. Of ten studies in Zafeiroudi et al.’s [3] review, only three employed mixed methods; most were purely quantitative or purely qualitative. This protocol’s simultaneous hypothesis-testing and mechanistic exploration is rare in recreational dance research.

This protocol includes comprehensive within-intervention assessment (T0, T1, T2 spanning all three motor learning phases) with weekly journaling documenting embodied progression. However, the protocol lacks post-intervention follow-up assessment beyond Week 12 to evaluate behavioral maintenance and sustained effects. While motor learning consolidation occurs within 6–12 weeks [14], generalization to daily life requires follow-up at 3, 6, 12 months post-intervention. Future research must employ extended follow-up protocols to establish sustainability, directly addressing Duberg et al.’s [60] evidence for maintained effects at 20 months.

While Marich & Howell [5] identified dance and music as core therapeutic mechanisms and Porges [8] emphasizes nervous system dysregulation as the underlying pathology, this protocol lacks direct psychophysiological assessment. The present protocol prioritizes accessibility and scalability (no biomarkers) at the expense of mechanistic clarity.

Christensen [1] documented substantial variation in dance modality effects (e.g., Argentine Tango reduces Parkinson symptoms; Waltz does not), suggesting that dance style is a critical moderating variable. This protocol specifies ISTD modern dance within Greek recreational settings. Generalizability to other dance styles, geographic contexts, socioeconomic populations, or cultural adaptations is limited [3,61]. Comparative research examining this protocol’s model against yoga and tai-chi and other mind–body movement is critical for understanding whether common pedagogical principles (phase-based nervous system scaffolding, motor learning optimization, somatic reflection) generalize across embodied traditions and should be added in future research.

The protocol’s structured design offers significant advantages for educational and community implementation. Universities could integrate this model into recreation curricula, repositioning dance toward comprehensive well-being practice. High schools could adapt it for health curricula. Community centers could deliver this protocol with modest facilitator training (12 h standardized module). Dance educators increasingly pursue MBSR teacher certification (intensive), yoga teacher training (200 h), or trauma-informed somatic training, credentials that naturally dovetail with this protocol. The protocol positions evidence-based embodied practice as implementable in understaffed community settings where clinical providers are unavailable, while maintaining rigor through explicit fidelity monitoring.

This evidence base, demonstrating that systematic nervous system development combined with motor learning, somatic reflection, and relational engagement produces measurable psychological improvements, carries implications extending beyond dance. Additionally, the integrative model could adapt for recreational yoga (sequencing poses across three phases: parasympathetic establishment, technical refinement, esthetic exploration), tai-chi and qigong (systematizing existing contemplative structure), and Pilates (operationalizing nervous system principles underlying practice). The contribution demonstrates that underlying pedagogical structures (phase-based nervous system scaffolding, motor learning optimization, somatic inquiry, relational attunement) may optimize outcomes across practices.

University wellness programs could implement this protocol with undergraduate peer educators, providing cost-effective, evidence-based intervention aligned with return-on-investment metrics (improved life satisfaction, reduced psychological distress, enhanced attention, increased leisure involvement translating into improved productivity and retention). For clinical and community mental health, this protocol demonstrates that recreational embodied practice produces measurable psychological benefits without clinical diagnosis framing, addressing Zhang and Wei’s [4] observation that movement-based approaches leverage mind–body connection but often lack rigorous evidence. This positions evidence-based embodied practice as prevention/well-being promotion complementing (not replacing) clinical treatment, with implications for health equity.

## 6. Conclusions

This protocol outlines a comprehensive, evidence-informed 12-week integrated dance-mindfulness intervention designed to systematically operationalize polyvagal theory, motor learning science, somatic education, and embodied phenomenology into coherent pedagogical practice. Grounded in principles of progressive nervous system development, technical skill optimization, and relational attunement, the proposed three-phase framework addresses a critical gap in recreational dance education by bridging abstract neuroscientific theory and concrete teaching methodology. The intervention responds to what recreational dancers authentically seek: nervous system resilience, embodied confidence, authentic creative expression, and social belonging—motivations that are largely absent from both clinical and elite professional dance research.

If supported by future data, this approach may offer a replicable and adaptable model for transforming recreational dance from technical skill development alone toward a comprehensive well-being practice grounded in contemporary neuroscience and embodied meaning-making. Beyond recreational dance, findings will inform contemplative movement education broadly (yoga, tai-chi, Pilates, qigong) and clarify scalability across educational, community, and clinical settings, with implications for integrating neuroscience-informed embodied practice into diverse organizational contexts. This protocol contributes to evidence-based pedagogical innovation by demonstrating that systematic integration of nervous system regulation, motor learning, somatic reflection, and relational engagement produces superior psychological and embodied outcomes compared to traditional dance instruction alone, fundamentally advancing how movement education serves adult psychological well-being and authentic self-expression.

## Figures and Tables

**Table 1:** Quantitative Outcome Measures.

Instrument	Acronym	Measure	Items	Response	Representative Items	Validation	Time Points
Satisfaction With Life Scale	SWLS	Subjective life satisfaction.	5 indicating participant’s degree of agreement.	7-point Likert scale (1 = Strongly Disagree to 7 = Strongly Agree)	“In most ways my life is close to ideal”; I am satisfied with my life.”	[33]	Baseline (T0) & post-intervention (T2).
Depression Anxiety Stress Scales-21	DASS-21	Negative emotional states by measuring levels of depression, anxiety, & stress.	21 distributed equally across three subscales (7 items per subscale measuring depression, anxiety, stress dimensions).	4-point Likert scale (1 = Did Not Apply to Me at All to 4 = Applied to Me Very Much or Most of the Time)	“I couldn’t feel any positive feelings” (depression subscale); “I felt I was close to panic” (anxiety subscale).	[34]	Baseline (T0) & post-intervention (T2).
Mindful Attention Awareness Scale	MAAS	Mindfulness, the capacity of participants to be attentive and fully present in the current moment [35].	15 describing everyday experiences of inattention.	6-point Likert scale (1 = Almost Always to 6 = Almost Never)	“I could experience some feeling and not be aware of it until some time later”; “I rush through activities without being really attentive to them.”	[36]	Baseline (T0) & post-intervention (T2).
Subjective Happiness Scale	SHS	Subjective happiness [37].	4 statements/questions.	7-point Likert scale	“In general, I consider myself: 1 = Not a very happy person … 7 = A very happy person”; “Compared to most of my peers, I consider myself: 1 = Less happy … 7 = Happier.”	[38]	Baseline (T0) & post-intervention (T2).
Leisure Involvement Scale	LIS	Psychological engagement & commitment of participants to a specific recreational activity.	13 examining dimensions, e.g., activity attractiveness/interest, centrality to the individual’s life, identification/identity through the activity.	7-point Likert scale (1 = Strongly Disagree to 7 = Strongly Agree)	“Participating in the activity I chose is one of the most captivating experiences”; “Participating in the activity I chose is one of the things that satisfies me most.”	[39]	Baseline (T0) & post-intervention (T2).

**Table 2:** Component 1: Warm-Up and Breath Grounding.

Element	Phase 1 (Weeks 1–4)	Phase 2 (Weeks 5–8)	Phase 3 (Weeks 9–12)
Duration	9–10 min	9–10 min	9–10 min
Motor Learning Stage	Cognitive	Associative	Automatic
Nervous System Focus	Parasympathetic foundation	Sympathetic–parasympathetic balance	Nervous system coherence
Breath Pattern	4-count nasal breathing	Coordinated breath-movement with elevated activity	Automatic nasal breathing during complex flow
Movement Complexity	Simple bilateral patterns	Multi-directional coordination	Unified continuous flow
Section Structure	4 sections & closing	4 sections & closing	3 sections (unified) & closing
Content & Movement	Foundational somatic awakening: synchronized 4-count nasal breathing with gentle arm/leg movements; light rhythmic activation (walking, side steps, soft bouncing); joint mobility (head, shoulder, arm, hip circles; standing spinal waves); dynamic stretching. Establishes embodied presence and nervous-system readiness [18,44].	Integration of breath-movement synchronization and respiratory–cardiac coupling awareness: walking, running, side steps, bouncing with coordinated arm–leg patterns at moderate tempo while maintaining breath coherence.	Continuous 7 min coherent flow linking breath, walking, running, bouncing, combination of directional steps, coordinated arm–leg patterns, spine and whole-body mobility; effortless rhythm emphasizing self-regulation and parasympathetic stability [8,14,45].
Mindfulness Practices	Foundational breath observation and sensory grounding. Facilitator cue: “Notice your breath, is it natural and easy? Focus on gentle awareness of respiration and contact with the floor.”	Coordination of breath with movement and interoceptive mapping, noticing heartbeat, warmth, and breath flow during dynamic transitions. Facilitator cue: “Feel your heartbeat and breath moving together, let movement ride your breathing rhythm.”	Sustained coherent breathing within continuous movement flow; attention expands to whole-body rhythmic unity and emotional steadiness. Facilitator cue: “Let breath and movement merge, effortless rhythm, full presence.”

**Table 3:** Component 2: Technical Dance Work.

Element	Phase 1 (Weeks 1–4)	Phase 2 (Weeks 5–8)	Phase 3 (Weeks 9–12)
ISTD Grade	Grade 2	Grade 3	Grade 4
Duration	14–15 min	14–15 min	14–15 min
Motor Learning Stage	Cognitive	Associative	Automatic
Primary Focus	Sensory awareness & body literacy.	Proprioceptive deepening.	Expression through mastery.
Nervous System Quality	Parasympathetic baseline during learning.	Sustained sympathetic engagement with support.	Coherent regulation through complexity.
Content & Technical Work	Foundational sensory awareness: basic Grade 2 exercises (side stretch, forward stretch, arm exercise) while developing body literacy, the ability to identify and articulate bodily sensations. Dancers learn basic patterns while optimizing motor synergies and reducing unnecessary muscle tension [46,47]. Movement structured around internal sensation rather than external form correction.	Proprioceptive deepening: Grade 3 complexity (enhanced side stretch, forward stretch depth, abdominal engagement) with emphasis on breath-guided transitions and subtle internal adjustments. Explicit and implicit motor sequence learning occur simultaneously; participants consciously recall movement sequences while implicit systems optimize motor efficiency and binding [12]. Facilitator cuing shifts from “correct positioning” to “what does your breath tell you about appropriate depth?”	Embodied expression: complex Grade 4 sequences (forward and side stretch, spine loosening, arm exercise) prioritizing authentic personal expression over mechanical precision. Technical skill becomes clear, serving the dancer’s authentic voice [23]. Transfer learning principles enable Grade 3 to Grade 4 compatibility through shared movement transitions [13].

**Table 4:** Component 3: Conditioning and Strength Work.

Element	Phase 1 (Weeks 1–4)	Phase 2 (Weeks 5–8)	Phase 3 (Weeks 9–12)
Duration	9–10 min	9–10 min	9–10 min
Motor Learning Stage	Cognitive	Associative	Automatic
Primary Focus	Embodied effort & permission-giving.	Nervous system oscillation & flexibility.	Vagal efficiency & sustained regulation.
Nervous System Quality	Parasympathetic foundation during activation.	Sympathetic engagement with parasympathetic support.	Coherent regulation through increasing intensity.
Content & Conditioning Work	Foundational embodied strength: basic exercises (half roll-downs, rolling like a ball, prone swimming) while developing conscious effort-awareness, the ability to sustain work while maintaining steady breath. Participants learn basic movement patterns while experiencing that activation need not trigger dysregulation [48]. Movement structured around internal breath-state rather than external performance metrics. Breath is the anchor. If breath becomes held or jagged, intensity is reduced.	Intermediate endurance: rhythmic lower-body work (barre footwork, dynamic stretching) emphasizing oscillation between effort and recovery. Participants consciously monitor breath to self-regulate intensity while implicit motor systems optimize efficiency [9]. Explicit regulation (breathing consciously) paired with implicit learning (body finds sustainable rhythm). Facilitator cuing shifts from “maintain effort” to “let your breath guide your intensity.” Nervous system learns: effort and calm are partners, not opponents.	Complex whole-body integration: upper-body resistance and plank variations prioritizing vagal efficiency. Meeting significant physical demand while maintaining nervous system regulation. Technical conditioning becomes clear, serving embodied resilience and neuroception shift [9,49]. Participants demonstrate capacity to sustain intensity without dysregulation; this lived experience retrains the nervous system’s threat-detection [48]. Transfer learning from Phase 2 enables increased Phase 3 intensity through maintained regulation [49].
Interoceptive Cues	Foundational effort-awareness. Facilitator cue: “Where do you feel effort? Your legs? Your core? Your breath? There’s no wrong answer, just notice. Breathe through the work, not against it. Your nervous system is learning: I can work and stay calm simultaneously.” Develops interoceptive capacity as foundation for embodied resilience.	Breath-guided self-regulation. Facilitator cue: “Notice your heartbeat is faster. Your breath is steady. Feel that: you’re activated and regulated. If your breath becomes jagged, slow down. Your breath is your guide, not a metric to chase. Your nervous system learns to oscillate between effort and recovery.” Integrates breath-awareness with nervous system flexibility.	Embodied resilience. Facilitator cue: “Meet this intensity with curiosity, not resistance. Your body is meeting significant demand. Your breath is steady. Your nervous system is regulated. Feel that capacity: I can sustain. I can persist. I can meet challenge with my whole self-present. This is embodied resilience. This is nervous system healing.” Embodies non-judgmental acceptance of intensity maintained regulation as pathway to resilience and flow in recreational practice [9].

**Table 5:** Component 4: Break/Transition and Nervous System Settling.

Element	Phase 1 (Weeks 1–4)	Phase 2 (Weeks 5–8)	Phase 3 (Weeks 9–12)
Duration	4–5 min	4–5 min	4–5 min
Primary Focus	Parasympathetic reactivation & downward self-regulation.	HRV restoration & sympathetic-parasympathetic oscillation.	Embodied belonging & collective co-regulation.
Nervous System Quality	Parasympathetic dominance re-established.	Parasympathetic reactivation evident (HR slowing, breath deepening).	Ventral vagal coherence & relational attunement.
Recovery Metric	Visible calm; steady breath; relaxed facial expression.	Heart rate approaching baseline; HRV improving [11].	Subjective sense of safety & felt sense of connection [10].
Content & Recovery Work	Foundational settling: gentle supine or seated positioning with interoceptive grounding, participants notice body weight settling into floor/chair, breath becoming audible and steady. Facilitator guides attention to: “Feel where your body contacts the surface beneath you… feel your weight releasing… feel your breath becoming calm and easy” [17]. No additional movement; stillness prioritized. Movement structured around parasympathetic anchoring rather than cognitive narrative. Develops embodied confidence: “My nervous system can settle. I am safe. Recovery is natural.”	Intermediate settling: seated or supine with gentle proprioceptive grounding. Facilitator emphasizes oscillation: “Notice: your heartbeat is slowing. Your breathing is deeper. Your nervous system is finding its way back to calm”. Participants practice HRV-aware settling, tracking visible signs of vagal reactivation (slowed heart rate, deeper breathing, muscle relaxation). Acknowledgment of recovery process: “This is your parasympathetic nervous system reactivating. This is your body’s natural return to safety.” Nervous system learns: oscillation is reliable; recovery is predictable.	Advanced settling: supine with collective presence. Facilitator emphasizes relational co-regulation: “You are settling. Notice others around you also settling. We are together in this calm. Feel that you belong in this shared safety”. Participants experience “feeling felt” through group nervous system coherence, a phenomenon where one person’s regulated state influences others’ autonomic nervous systems [10,17]. Recovery metric shifts from individual interoception to relational attunement. “Sense of self” (SPA: sensation, perspective, agency) integrates with “sense of we” through co-regulated presence.
Interoceptive Cues	Foundational settling-awareness. Facilitator cue: “Feel your weight releasing into the earth/chair. Your nervous system is learning to settle. Notice: there’s no danger here. There’s nowhere to go. There’s nothing to do. Just settle. Just be. Your body knows how to rest.”. Develops parasympathetic confidence through gentle proprioceptive anchoring [17].	Oscillation-aware recovery. Facilitator cue: “Your heartbeat is slowing down. Count if you like: how many beats slower than 30 s ago? Your breath is deepening. Feel the ease returning to your belly, your shoulders, your jaw. Your nervous system is oscillating back to parasympathetic safety. This is recovery. Trust it”. Integrates explicit HRV awareness with somatic sensation [11,50].	Relational settling & belonging. Facilitator cue: “You are safe. Others are safe. We are together in this calm. Look around if your eyes are open, notice faces relaxed, bodies settled. Feel that collective safety. This is what belonging feels like, our nervous systems together, regulated and calm. You are not alone in this settling. We settle together”. Embodies interpersonal neurobiology: individual mental health arising through relational connection and shared nervous system coherence [10].

**Table 6:** Component 5: Rhythm and Improvisation.

Element	Phase 1 (Weeks 1–4)	Phase 2 (Weeks 5–8)	Phase 3 (Weeks 9–12)
Duration	8–9 min	8–9 min	8–9 min
Primary Focus	Rhythm foundations & structured novelty.	Guided improvisation & flow conditions.	Full absorption & optimal creativity.
Flow Preconditions	Moderate challenge, moderate skill, clear rhythm as “goal”.	Challenge-skill balance at moderate-high levels.	Optimal challenge-skill match, high intrinsic motivation.
Nervous System Quality	Parasympathetic baseline maintained despite novel rhythm.	Sympathetic engagement & parasympathetic access (flow window).	Dopaminergic activation (drive, pleasure) & noradrenergic focus (task engagement).
Content & Rhythm-Improvisation Work	Foundational rhythm: basic pulse awareness through walking/stepping to steady beat; minimal variation (left-right, forward-back) within structured frame. Participants experience novelty through rhythm (not through complex choreography), building intrinsic motivation through autonomy (“You choose whether to step or bounce to this beat”) within contained parameters. Clear “challenge means matching rhythm externally” and skill means listening to pulse.” Balance is accessible; participants maintain felt sense of competence. Music: steady most 4/4 beat, predictable tempo.	Intermediate improvisation: guided explorations of rhythm using given movement vocabulary (steps, bounces, arm patterns established in Components 1–3). Facilitator provides rhythmic structure but invites participant choice: “Use these movements to respond to the music” (autonomy). Challenge increases through tempo variation; skill increases through familiarity with vocabulary. Flow state conditions emerge: participants absorbed in rhythm–movement matching, experiencing merging of action-awareness, time distortion [20]. Loss of self-consciousness occurs; individuals move authentically rather than self-consciously. Music: varied tempos, polyrhythmic layers invite adaptive response. Dopamine system activated through novelty within mastery [51].	Advanced improvisation: full creative autonomy within dance–rhythm framework. Participants generate novel movement responses to music in real-time. Challenge maximized through musical complexity and creative demands; skill maximized through 8 weeks of embodied preparation. Optimal skill-challenge balance achieved. Flow becomes dominant state: full absorption, loss of self-consciousness, time distortion, lack of concern about evaluation [20,51]. Dopaminergic reward systems activated through intrinsic satisfaction; noradrenergic system sustains task engagement through continuous feedback (self-generated through proprioceptive awareness). Improvisation serves as anxiety-reduction [22] and embodied authenticity pathway. Participants access autotelic experience—a state that is pleasurable in itself and intrinsically valued [51].
Interoceptive Cues	Rhythm-awareness grounding. Facilitator cue: “Feel the pulse. Let your feet follow the beat. Can you feel your heartbeat matching the rhythm? You’re in control, step when it feels right. Notice: the music, your body, your choices.”. Develops intrinsic motivation through autonomy & competence balance [51].	Flow-inducing absorption. Facilitator cue: “You’ve learned these movements. Now improvise. Use them to respond to what you hear. Don’t think, feel the rhythm and move. Notice when you’re fully absorbed. Notice when you forget to judge yourself. That’s flow”. Introduces explicit flow language; participants become aware of flow state as it emerges [20].	Embodied creativity & authenticity. Facilitator cue: “This is your dance. The rhythm is the invitation. Your body is the answer. Move authentically, not how you think you should move, but how your body wants to respond to this music. You are fully here. Fully expressed. Fully alive”. Embodies intrinsic motivation, embodied authenticity, and somatic knowledge acquired through 8 weeks of integrated practice [21,22]. Merges personal agency with collective rhythm.

**Table 7:** Component 6: Dance Sequences and Choreographed Material.

Element	Phase 1 (Weeks 1–4)	Phase 2 (Weeks 5–8)	Phase 3 (Weeks 9–12)
Duration	15–17 min	15–17 min	15–17 min
Primary Focus	Explicit motor sequence learning & individual choreographic competence.	Implicit consolidation & small-group ensemble synchronization.	Automaticity, embodied authenticity & collective flow.
Motor Learning Phase	Cognitive phase: explicit, declarative knowledge acquisition [12].	Associative phase: binding elements into skilled behavior; implicit learning emerging [12].	Automatic phase: somatic knowledge, second nature, embodied transformation [12,52].
Nervous System Quality	Beta oscillations active in motor cortex (early learning); sympathetic engagement & parasympathetic access maintained through breath-grounding cues.	Beta power increases (consolidation); inter-brain synchronization emerging; collective parasympathetic stability.	Motor cortex efficiency; dopaminergic reward and intrinsic motivation [15]; ventral vagal coherence in ensemble coordination [9].
Choreographic Content & Learning Work	Foundation sequence A & B (Grade 2): step ball-changes, basic turns, simple combinations (4–5 min learning & 4–5 min practice). Individual focus: participants develop declarative knowledge of sequence steps, timing, rhythm alignment [12]. Movement remains cognitively demanding; facilitator provides explicit cues: “Right foot, ball of foot, together. Left foot, ball of foot, together.” Participants attend consciously to each element. Music: mostly 4/4, predictable phrasing. Breath anchored throughout: facilitator cue: “Breathe through the step ball-changes. Your breath makes space in your chest for the movement.” Individual competence means sequence learned step-by-step; participants experience early-learning effortfulness mastered.	Intermediate sequence (Grade 3): triple runs, turns with directional changes, small group pairings/trios (3–4 min learning & 4–5 min ensemble practice). Implicit learning accelerates: participants move with less conscious attention to individual steps; focus shifts to relationship (with partner or small group). Facilitator introduces ensemble awareness (operationalized from [9]: “Your partner is breathing too. Your heartbeat synchronizes with theirs. Move together, not just side-by-side.” This operationalizes polyvagal theory mechanisms of co-regulation and social engagement. Inter-brain synchronization begins as nervous systems resonate. Small groups practice sequence together; individual skill supported by group rhythm. Movement becomes more automatic; effort decreases. Music: varied phrasing, relational themes. Facilitator observation: Watch for moments when participants stop watching each other and begin feeling each other’s movement, that’s implicit ensemble learning emerging.	Advanced sequence: full integrated sequence with ensemble spatial patterns, level changes, counterpoint (5–7 min ensemble flow & 2–3 min reflection). Automaticity permits authentic relational presence. Individual somatic knowledge is now embodied; movement requires minimal conscious attention. Attention available for connection, genuine eye contact, responsive timing, felt sense of collective coherence [15]. Ensemble synchronization is not forced; it emerges from automaticity & embodied mindfulness. Participants experience: merging of action-awareness (their movement and others’ movement blur into collective flow); loss of self-consciousness (no performance anxiety, no judgment); time distortion (sequence feels simultaneous and eternal). Facilitator invitation: “The choreography is now yours. It’s not being performed; it’s being lived. Feel the collective intelligence in this room. This is ensemble.” Movement embodies seven attitudinal factors: non-judging (accepting differences in timing), patience (allowing ensemble to gel), beginner’s mind (discovering sequence anew each time), trust (in partners, in collective), non-striving (flowing rather than forcing), acceptance (of what emerges), letting go (of perfectionism). Optimal skill-challenge balance achieved; flow state dominant [15].
Interoceptive Cues & Facilitator Language	Foundational explicit learning. Facilitator cue: “Step ball-change: right foot steps on the ball, immediately left foot steps on the ball, together. Right. Left. Together. Feel your weight shifting. Your breath matches the rhythm. One step-ball-change per breath phrase.” Emphasis on conscious attention to steps; participants developing explicit memory of sequence [13].	Intermediate ensemble awareness. Facilitator cue: “You know this sequence now. Your body has learned it. Now I’m inviting you to feel your partner’s body next to yours. Are they breathing? Are they grounded? Match your timing to their heartbeat. You’re two nervous systems breathing together.” Shift from cognitive to relational attention [9].	Advanced embodied authenticity. Facilitator cue: “This sequence is in your body now. You don’t need to think it. Your body knows. What’s available now is presence, genuine presence with each other. Move authentically, not how you think you should. Feel the collective intelligence. We are together, not trying to be together. We simply are.” Operationalizes flow state [15], non-striving [52], and collective coherence [9]. Emergence of felt sense of ensemble belonging and embodied authenticity.

**Table 8:** Component 7: Cool-Down and Reflective Integration.

Element	Phase 1 (Weeks 1–4)	Phase 2 (Weeks 5–8)	Phase 3 (Weeks 9–12)
Duration	8–9 min	8–9 min	8–9 min
Primary Focus	Basic interoceptive awareness & parasympathetic settling via body scan.	Expanded stretching & reflective embodied awareness.	Full mindfulness integration & embodied authenticity in reflection.
Memory Consolidation Quality	Synaptic consolidation: sensations encoded via systematic body scan attention [53].	Systems-level consolidation: hippocampal-cortical dialog through reflective body awareness [53].	Declarative & implicit memory consolidation: somatic knowledge integrated with narrative meaning-making [10].
Embodied Reflection Framework	Foundational body scan: participants develop “sixth sense” through interoceptive attending; basic vertical integration [10].	Intermediate embodied reflection: body phenomenology approach; attending to body-world relations; noticing hidden assumptions through embodied sensation [52,54].	Advanced embodied authenticity: body as meeting place of consciousness & world; seven attitudinal factors embedded in reflective practice; full vertical & bilateral integration [10,54].
Cool-Down & Stretching Work	Section 1 (1–1.5 min): Walking in place, gentle arm circles, deceleration to stillness. Section 2 (2–2.5 min): Upper back, leg, shoulder rolling, basic stretching. Section 3 (2–2.5 min): Guided body scan (supine or seated), attention to physical sensations from feet to crown. Closing (30–40 sec): Basic nervous system acknowledgment (“You’ve worked, now you rest”). Total: 8–9 min. Movement: slow, gentle, parasympathetic-supportive. Interoceptive focus: physical sensation only [52].	Section 1 (1–1.5 min): Slow walking with arm mobility (circles, swings, reaches). Movement remains mindful, continuous. Section 2 (2–3 min): Deeper static stretching, shoulder rolls, chest openers, forward fold, hip stretches, spinal twist, neck stretches. Facilitator language: “Feel what’s being released. What sensations appear as you stretch?”. Section 3 (2–2.5 min): Seated or supine body scan with reflective embedding, participants notice sensations and emotions simultaneously. Facilitator cue: “What’s present? Warmth? Tingling? Satisfaction? Calm? Just notice”. Section 4 (1 min): Reflective integration, participants notice what they’ve learned about their body today [10,53]. Total: 8–9 min.	Section 1 (1 min): Mindful walking with full presence, participants experience embodied authenticity through slow, conscious movement. No directive; facilitator models’ receptivity. Section 2 (3–3.5 min): Full dynamic & static stretching sequence. Facilitator integrates seven attitudinal factors: “Non-judge what appears. Stretch with patience, not forcing. Approach your body with beginner’s mind. Trust its intelligence. Non-strive; let release unfold. Accept what you find. Let go of what no longer serves” [52]. Section 3 (2.5–3 min): Extended body scan (full body, systematic attention) with invitation to notice body–mind emotion unity. Facilitator: “Scan your feet, they’ve carried you through joy and challenge. Scan your legs, they’ve rooted you. Scan your belly, your center. Scan your heart, it’s been open today. Scan your mind, it’s been present. Feel yourself whole”. Section 4 (1–1.5 min): Reflective closure with meaning-making. Facilitator: “What did your body teach you today? What did you learn about yourself through movement? Carry that knowing forward”. Total: 8–9 min.
Interoceptive Cues & Facilitator Language	Foundational body scan cue. Facilitator: “Close your eyes. Feel your feet, they’ve moved today. Feel your legs, they’ve worked. Move to your belly, breath flows here. Move to your heart, it’s beating steadily. Move to your shoulders, they’re releasing. Move to your face, it’s soft. Your whole body is supported, safe, at rest”. Emphasis on physical sensation without emotional interpretation [52].	Intermediate reflective awareness. Facilitator: “As you stretch, notice: What’s being released? What sensations appear? Tingling? Warmth? Relief? And also notice: What emotion is present? Gratitude? Calm? Satisfaction? Your body holds stories. Your stretches are releasing them”. Shift from sensory to sensory & emotional awareness [54]. Embodied reflection framework active.	Advanced embodied authenticity. Facilitator: “Your body is your teacher. Everything it needs to know about presence, acceptance, and letting go, it already knows. As you move and stretch and scan, practice the seven attitudes: non-judging (notice without critique), patience (let release unfold), beginner’s mind (approach anew), trust (in your body’s intelligence), non-striving (no forcing), acceptance (of what is), letting go (of what was). Feel how these attitudes live in your body now” [10,52,54].

**Table 9:** Component 8: Phenomenological Journaling.

Element	Phase 1 (Weeks 1–4)	Phase 2 (Weeks 5–8)	Phase 3 (Weeks 9–12)
Duration	10–15 min	12–17 min	14–20 min
Primary Focus	Foundational narrative-emotional meaning: Individual nervous system learning.	Deeper narrative-emotion integration: Relational awareness & emotional authenticity.	Full phenomenological integration: Four-dimensional transformation (individual, relational, spatial, social).
Narrative-Emotion Process Stage	Inchoate Storytelling emerging: tentative articulation of new embodied experience [56]; moving from “I did steps” toward “I discovered my capacity”.	Transition Markers active: Competing Plotlines (“I thought I was shy, but I moved freely”) & Reflective Storytelling (“I notice my nervous system is changing”) leading toward Discovery Storytelling [56].	Discovery Storytelling dominant: coherent, integrated narratives of personal & relational transformation; embodied reflective practice fully articulated [25,57].
Re-Representation & Memory Consolidation	Consolidation begins: embodied sensorimotor experience translated into language (re-representation); implicit learning becomes explicit narrative [55].	Consolidation deepens: emotion-meaning integration; participants recognize patterns across weeks; implicit emotional awareness becomes articulated feeling-meaning [58].	Consolidation complete: four-dimensional learning re-represented as coherent personal narrative; implicit & explicit learning systems integrated [55,58].
Journaling Content & Process	Phase 1 Structure (10–15 min total): Opening (1–2 min): “Find a comfortable place to sit. Take a pen and paper. We’ve moved together for 70 min. Now we invite your mind to articulate what your body has learned”. Prompts (8–10 min): Facilitator reads 2–3 of the following: (1) “What did your body discover today?”, (2) “Where did you feel most alive?”, (3) “What surprised you about yourself in movement?”, and (4) “What did you learn about your nervous system?” Participants write freely, not answering all prompts, just following what resonates. Closing (1–2 min): “Set your pen down. You’ve documented your individual learning journey. Your words are evidence of your transformation”.	Phase 2 Structure (12–17 min total): Opening (1–2 min): “Today we invite you to write about what you’ve learned about yourself and about connection. How is your body changing in relation to others? What are you discovering about connection, about belonging?”. Prompts (9–13 min): Facilitator reads 3–4 of the following: (1) “What did you feel moving alongside others today?”, (2) “Where did you experience connection? With your body? With others? With the space?”, (3) “What patterns are you noticing across these weeks of practice?”, (4) “What nervous system shift have you noticed in yourself? What evidence do you have?”, and (5) “What surprised you about your own presence or authenticity today?” Participants write to 2–3 prompts, following internal resonance. Facilitator model’s reflective stance: “There’s no right answer. Your authentic writing is your data”. Closing (1–2 min): “Your words are becoming evidence of your transformation from individual work to relational presence. That’s essential learning”.	Phase 3 Structure (14–20 min total): Opening (2–3 min): “We’re at the end of 12 weeks. Today we invite you to write about your complete transformation across four dimensions: your individual nervous system, your relational attunement, your inhabitation of space, your sense of belonging to community. How have you transformed across all four? Write your authentic truth”; Prompts (10–14 min): Facilitator reads 4–5 of the following (participants choose which to write to): (1) “Individual Dimension: How has your nervous system transformed? What evidence of regulation, authenticity, embodied presence do you notice?”, (2) “Relational Dimension: How has your capacity for connection shifted? What have you learned about moving with others? About feeling felt?”, (3) “Spatial Dimension: How do you inhabit space now? What’s different about your relationship to the room, to the ground, to your body’s presence in space?”, (4) “Social Belonging Dimension: What do you feel when you think of ‘we’, this community? How has belonging changed your sense of self?”, and (5) “Integration: What is the relationship between these four dimensions? How do they support one another?” Participants write continuously, following their own pace. Facilitator creates sacred silence: “I’m here. Your words matter. Take your time”. Closing (3–5 min): Comprehensive integration cue [57,58].
Embodied Reflective Practice Framework	Foundational embodied reflection: participants attend to body sensations translated into words; esthetic experience (movement) beginning to become narrative (language) [25].	Intermediate embodied reflection: participants integrate sensory & emotional & relational dimensions; metaphorical language emerges (“I felt like I was dancing with my fear and it softened”); esthetic experience becoming embodied narrative [57].	Advanced embodied reflective practice: participants articulate four-dimensional transformation; metaphorical language integrated with conceptual clarity; esthetic experience fully becomes coherent personal narrative & meaning-making; participants recognize themselves as transformed agents in community [25,58]
Facilitator Stance & Language	Witnessing presence. Facilitator language: “Your writing doesn’t need to be perfect. Your authentic articulation is all that matters. What emerges on the page is your truth”. Role: Read prompts with presence, create safe silence, witness writing without observation, receive completed journals with respect [55].	Embodied facilitation & narrative witnessing. Facilitator language: “As you write, feel your body. Notice what wants to be said. Your words are consolidating your learning. Your narrative is your evidence of change”. Role: Hold space for emotional-narrative emergence, normalize competing narratives (“It’s okay if you feel confused sometimes; that’s discovery”), receive writings with professional reverence [56].	Sacred witnessing & transformation acknowledgment. Facilitator language: “You’re writing your own transformation story. This is sacred work. Your journal is your evidence. Your body is living proof. Your presence in community is ongoing practice”. Role: Create altar-like container for reflection, read prompts with ritualistic presence, maintain profound silence during writing, and receive completed journals as sacred artifacts. Optional: Participants may share one sentence with a partner/group if desired (never forced) [25,58].

## Data Availability

The data presented in this study are available upon request from the corresponding author, due to ethical and privacy reasons.

## Supplementary Materials

The following supporting information can be downloaded at: https://doi.org/10.5281/zenodo.18713796 [27], Supplementary File S1: Intervention Protocol Manual; Supplementary File S2: Facilitator Guidelines and Safety Standard Operating Procedures (SOP); Supplementary File S3: Journaling Prompts & Interview Guide. Qualitative Coding Framework; Supplementary File S4: Fidelity Tools; Supplementary File S5: Consent Form; Supplementary File S6: TIDieR Checklist; Supplementary File S7: SPIRIT Checklist; Supplementary File S8: Ethics Committee Approval Documentation.

## Abbreviations

The following abbreviations are used in this manuscript:

CONSORT	Consolidated Standards of Reporting Trials
CPR	Cardiopulmonary Resuscitation
DASS-21	Depression Anxiety Stress Scales-21
dB	Decibels (sound level)
DPESS	Department of Physical Education and Sport Science
GDPR	General Data Protection Regulation
Hz	Hertz (frequency)
ICC	Intraclass Correlation Coefficient
IEC	Internal Ethics Committee
IRB	Institutional Review Board
IPA	Interpretative Phenomenological Analysis
ISTD	Imperial Society of Teachers of Dancing
LIS	Leisure Involvement Scale
lux	Lux (illumination measurement)
MAAS	Mindful Attention Awareness Scale
MBSR	Mindfulness-Based Stress Reduction
P1	Phase 1 (Weeks 1–4)
P2	Phase 2 (Weeks 5–8)
P3	Phase 3 (Weeks 9–12)
PI	Principle Investigator
PVT	Polyvagal Theory
RCT	Randomized Controlled Trial
RTA	Reflexive Thematic Analysis
S1–S8	Supplementary Files S1–S8
SHS	Subjective Happiness Scale
SPA	Sensation, Perspective, Agency
SPIRIT	Standard Protocol Items: Recommendations for Interventional Trials
SWLS	Satisfaction With Life Scale
T0	Baseline assessment (Week 0)
T1	Mid-intervention assessment (Week 8)
T2	Post-intervention assessment (Week 12)
TIDieR	Template for Intervention Description and Replication
UTH	University of Thessaly
